# Pilot clinical project: Trauma-Sensitive Yoga group intervention in psychiatric care

**DOI:** 10.1192/j.eurpsy.2026.11883

**Published:** 2026-07-31

**Authors:** L. V. A. C. Borges, C. D. García, A. I. Navarro

**Affiliations:** 1 CSM Puente de Vallecas; 2 CSM Villa de Vallecas, Hospital Universitario Infanta Leonor, Madrid, Spain

## Abstract

**Introduction:**

Post-traumatic stress disorder (PTSD) and complex PTSD (C-PTSD) are debilitating conditions that can develop after experiencing trauma and are characterized by intrusive symptoms, dissociation, hypervigilance, avoidance behaviors and persistent negative emotional states. C-PTSD is often the result of prolonged, repeated trauma and includes difficulties with emotional regulation, a negative self-image, and relationship problems.

Trauma-Sensitive Yoga (TSY) is a specialized practice designed for individuals who have experienced trauma. Unlike traditional yoga, TSY prioritizes safety, personal autonomy, interoception and nervous system regulation, while avoiding potential traumatic triggers, which allows participants to rebuild a sense of trust with their bodies. Research has demonstrated that yoga, and specifically TSY, can be a valuable tool for those suffering from PTSD. Studies have shown that it can significantly reduce symptoms such as hypervigilance, anxiety, and dissociation, while also improving emotional regulation.

**Objectives:**

To describe a pilot project that aims to implement a 10-session TSY in a small group of patients with PTSD/complex PTSD and to explore feasibility, acceptability, and preliminary changes in PTSD symptomatology.

**Methods:**

The participants are patients from a psychiatric outpatient service with a diagnosis of PTSD/C-PTSD or significant traumatic history with anxiety, depressive or dissociative symptoms. Exclusion criteria include severe symptoms preventing group participation, active substance use that interferes with the process, active suicidal ideation/risk and relevant medical conditions contraindicating physical exercise.

The intervention consists of weekly 60-minute TSY sessions over 10 weeks, co-facilitated by a psychiatrist trained in TYS and a psychologist. Each session addresses core components: safety, choice, present-moment awareness, interoceptive/proprioceptive awareness, autonomic regulation, and compassion. Sessions are followed by brief group reflection.

Participants will complete the Posttraumatic Stress Disorder Symptom Severity Scale-Revised (EGS-R) and the PTSD Checklist for DSM-5 (PCL-5) pre- and post-intervention. Clinical interviews are conducted to identify individual goals and evaluate subjective experiences.

**Results:**

At the time of submission, the intervention and data collection is ongoing. Descriptive feasibility outcomes (recruitment, attendance, adherence) and preliminary symptom changes will be presented.

**Conclusions:**

This pilot project will help us understand the feasibility and clinical value of TSY in a psychiatric setting. We hypothesize that TSY may represent a valuable complementary practice for patients with PTSD, supporting both symptom reduction and overall well-being.

**Disclosure of Interest:**

None Declared

